# Inflammation, coronary plaque progression, and statin use: A secondary analysis of the Risk Stratification with Image Guidance of HMG CoA Reductase Inhibitor Therapy (RIGHT) study

**DOI:** 10.1002/clc.23808

**Published:** 2022-04-02

**Authors:** Colin Scott, Sundus S. Lateef, Christin G. Hong, Amit K. Dey, Grigory A. Manyak, Nidhi H. Patel, Wunan Zhou, Alexander V. Sorokin, Khaled Abdelrahman, Domingo Uceda, Meron Teklu, Colin Wu, Philip M. Parel, Veit Sandfort, Marcus Y. Chen, Marissa Mallek, Mark Ahlman, David Bluemke, Nehal N. Mehta

**Affiliations:** ^1^ Section of Inflammation and Cardiometabolic Diseases, National Heart, Lung, and Blood Institute National Institutes of Health Bethesda Maryland USA; ^2^ University of Wisconsin Madison Wisconsin USA

**Keywords:** coronary calcification, inflammation, statin treatment

## Abstract

**Background:**

Statin treatment is a potent lipid‐lowering therapy associated with decreased cardiovascular risk and mortality. Recent studies including the PARADIGM trial have demonstrated the impact of statins on promoting calcified coronary plaque.

**Hypothesis:**

The degree of systemic inflammation impacts the amount of increase in coronary plaque calcification over 2 years of statin treatment.

**Methods:**

A subgroup of 142 participants was analyzed from the Risk Stratification with Image Guidance of HMG CoA Reductase Inhibitor Therapy (RIGHT) study (NCT01212900), who were on statin treatment and underwent cardiac computed tomography angiography (CCTA) at baseline and 2‐year follow‐up. This cohort was stratified by baseline median levels of high‐sensitivity hs‐CRP and analyzed with linear regressions using Stata‐17 (StataCorp).

**Results:**

In the high versus low hs‐CRP group, patients with higher baseline median hs‐CRP had increased BMI (median [IQR]; 29 [27–31] vs. 27 [24–28]; *p* < .001), hypertension (59% vs. 41%; *p* = .03), and LDL‐C levels (97 [77–113] vs. 87 [75–97] mg/dl; *p* = .01). After 2 years of statin treatment, the high hs‐CRP group had significant increase in dense‐calcified coronary burden versus the low hs‐CRP group (1.27 vs. 0.32 mm^2^ [100×]; *p* = .02), beyond adjustment (*β* = .2; *p* = .03).

**Conclusions:**

Statin treatment over 2 years associated with a significant increase in coronary calcification in patients with higher systemic inflammation, as measured by hs‐CRP. These findings suggest that systemic inflammation plays a role in coronary calcification and further studies should be performed to better elucidate these findings.

## 1 INTRODUCTION

Inflammation is vital to the progression of atherosclerosis and accounts for 20%–30% of residual risk for adverse cardiovascular events, driven in part by rupture of unstable coronary plaque.^1^, ^2^, ^3^ Systemic inflammatory diseases are associated with elevated risk of atherosclerotic events and premature cardiovascular disease.^4^, ^5^, ^6^, ^7^ In particular, patients with psoriasis, a chronic inflammatory disease, have an increased development in noncalcified coronary burden (NCB), which serves as an important predictor of future cardiac events beyond traditional risk factors.^8^, ^9^

Calcified plaques, commonly referred to as dense‐calcified coronary burden (DCB), are traditionally known to be more stable than NCB.^10^ Recently, the PARADIGM study^11^ reported that statin treatment promotes coronary artery calcification, thus conferring a lower risk of adverse cardiac events.^12^ Chronic inflammatory conditions, such as psoriasis, are considered a major indication to begin statin treatment in patients at intermediate risk of heart attack or stroke by the 2018 AHA Cholesterol Guidelines.^13^ Statins also reduce systemic inflammation as assessed by aortic FDG uptake on PET/CT.^14^ Further, they reduce incident cardiovascular events in those with elevated high‐sensitivity C‐reactive protein (hs‐CRP).^15^ Hs‐CRP is a prognostic biomarker of inflammation that predicts incident MI, stroke, and peripheral arterial disease.^16^ However, there have been limited efforts to determine whether the degree of systemic inflammation impacts the efficacy of statin treatment.

We sought to determine whether study participants at a higher baseline inflammatory state would derive greater benefit from statin treatment in stabilizing coronary plaque morphology than those with low systemic inflammation. To our knowledge, there is no study to date of this kind documenting varied plaque progression in patients with a high inflammatory state.

## 2 METHODS

### 2.1 Study participants

Study participants were a sub‐group from the Risk Stratification with Image Guidance of HMG CoA Reductase Inhibitor Therapy (RIGHT) study (NCT01212900), which was a 2‐year prospective, longitudinal cohort study of statin treatment that had 230 enrolled participants who were aged 55 years and older and recruited at the National Institutes of Health (NIH) Clinical Center (CC). Of these 230 participants, 180 patients fully completed the study with readable magnetic resonance imaging (MRI) scans at baseline and 2‐year follow‐up (Figure S1).

Participants either received statin treatment using an image‐guided assessment of atherosclerosis via coronary computed tomography angiography (CCTA), or statin treatment in accordance with standard clinical practice as described by the NCEP (National Cholesterol Education Program, Panel III) guidelines. After a 2‐year follow‐up, the two groups were compared for differences in carotid wall thickness. These primary results are reported on Clinicaltrials.gov (NCT01212900).

Our subcohort consisted of 142 participants who completed two sets of CCTA scans, in addition to the aforementioned MRI, to assess differences in coronary plaque progression and were further stratified based on the median value of baseline hs‐CRP (Table 1 and Figure S1).

**Table 1:** Baseline and 2‐year change characteristics of cohort stratified by median value of baseline hs‐CRP

Variable	Entire cohort (*n* = 142)	Low hs‐CRP (*n* = 76)	High hs‐CRP (*n* = 66)	*p*
**Demographic and clinical characteristics**				
Age (years)	65 ± 6.3*	64.7 ± 6.0	65.3 ± 6.8	.57
Males	92 (64)	53 (70)	39 (59)	.19
Hypertension	70 (49)	31 (41)	39 (59)	**.03**
Diabetes mellitus	10 (7)	4 (5)	6 (9)	.37
Body mass index	27 (25–29)	27 (24–28)	29 (27–31)	**<.001**
Current smoker	7 (5)	3 (4)	4 (6)	.59
Framingham Risk Score	7.3 (3.1–12.7)	7.1 (2.9–11.8)	8.2 (3.2–14.3)	.19
**Clinical and lab values**				
Total cholesterol, mg/dl	177 (154–199)	171 (148–188)	181 (159–202)	.06
*Delta (2‐year)*	*−14 (−40 to 4)*	*−12 (−29 to 6)*	*−21 (−53 to 1)*	*.05*
HDL cholesterol, mg/dl^a^	57 (46–70)	58 (48–75)	57 (45–69)	.26
*Delta (2‐year)*	*2 (−5 to 7)*	*3 (−4 to 10)*	*1 (−5 to 5)*	*.16*
LDL cholesterol, mg/dl^a^	90 (76–105)	87 (75–97)	97 (77–113)	**.01**
*Delta (2‐year)*	*−15 (−29 to −1)*	*−13 (−25 to −2)*	*−18 (−35 to −1)*	*.10*
Triglycerides, mg/dl	107 (72–138)	96 (67–121)	118 (78–149)	**.03**
*Delta (2‐Year)*	*−9 (−32 to 13)*	*−10 (−32 to 10)*	*−8 (−27 to 14)*	*.79*
CAC score	65 (1–380)	54 (1–320)	67 (0–467)	.98
*Delta (2‐year)*	*13 (0*–*80)*	*12 (1*–*78)*	*14 (0*–*90)*	*.82*
hs‐CRP, mg/L^a^	1.0 (0.5–2.1)	0.5 (0.3–0.8)	2.2 (1.6–4.2)	**.00**
*Delta (2‐year)*	*0.05 (−0.4 to 0.51)*	*0.16 (−0.05 to 0.4)*	*−0.43 (−2.31 to 0.64)*	***.001***
log (hs‐CRP)	−0.01 (−0.76 to 0.76)	−0.65 (−1.2 to −0.29)	0.77 (0.44–1.44)	**<.001**
*Delta (2‐year)*	*0.10 (−0.41 to 0.63)*	*0.36 (−0.11 to 0.75)*	*−0.30 (−0.84 to 0.33)*	***<.001***
* **Coronary artery characterization**				
Noncalcified coronary burden, (mm^2^×100)	3.5 (0.2–14.1)	3.3 (0.4–14.0)	3.6 (0.1–15.4)	.99
*Delta (2‐year)*, (mm^2^×100)	*0.17 (−0.41to 2.27)*	*0.24 (−0.44 to 2.28)*	*0.06 (−0.07 to 2.27)*	*.87*
Dense‐calcified coronary burden, (mm^2^×100)	6.5 (0.22–37.2)	6.5 (0.5–37)	6.1 (0–37.2)	.84
*Delta (2‐Year)*, (mm^2^×100)	*0.90 (−0.33 to 5.26)*	*0.32 (−0.08 to 2.50)*	*1.27 (0–9.80)*	***.02***
**Carotid artery characterization**				
Carotid artery thickness (mm^3^)	127 (109–148)	128 (110–146)	126 (102–153)	.65
*Delta (2‐year)*	*−4.96 (−17.9 to 7.88)*	*−2.38 (−19.3 to 7.88)*	*−6.13 (−14.9 to 11.0)*	*.94*

* Values are mean ± *SD* or median (IQR) for continuous data and *N* (%) for categorical data. Bolded *p * values are significant.

^a^ HDL cholesterol: high‐density lipoprotein cholesterol; LDL cholesterol: low‐density lipoprotein cholesterol; hs‐CRP: high sensitivity C‐reactive protein.

### 2.2 Inclusion/exclusion criteria

Exclusion criteria were ineligibility for MRI due to: previous pacemaker implantation, presence of automatic implantable cardioverter‐defibrillator, metal implants, or other ferromagnetic devices, and foreign material. Other exclusion factors included contraindication or allergy to statin medications, claustrophobia, current statin treatment at or above the maximum dosage permitted by study therapy, use of fibrates, ezetimibe, niacin, or bile acid binding agents within 6 months of screening visit, and pregnancy and nursing. All participants were provided written, informed consent, and all study protocols were approved by the institutional review board of the National Institutes of Health and complied with the Declaration of Helsinki.

### 2.3 Carotid artery wall volume measured by MRI

Carotid MRI was carried out using a 3‐T scanner and surface carotid coils. Images were acquired cross‐sectionally with a DIR fast spin‐echo pulse sequence, ECG gated, with black blood and fat suppression. Slice thickness was 2.0 mm with in‐plane resolution of 500–600 µm. Readers used commercially available contouring software (QPlaque, Medis Inc) and were blinded to group assignment.

### 2.4 Coronary burden characterization by coronary computed tomography angiography

All participants underwent CCTA using the same scanner (320‐detecter row Aquilion ONE ViSION). Guidelines implemented by the NIH Radiation Exposure Committee were adhered to. Scans were performed with retrospective gating at 120 kV, tube current of 750–850 mA, and a gantry rotation time of less than or equal to 420 ms. Coronary artery characteristics across the main coronary arteries greater than 2 mm diameter were analyzed using the dedicated software QAngio CT (Medis) with high intraclass correlation coefficient (>0.95). Only clear deviations of the software's automatic contouring of the outer wall and lumen segmentation were edited. Coronary artery segmental plaque area was indexed by length of the vessel to account for variable coronary artery lengths between participants and subsequently adjusted for mean luminal intensity to yield NCB and DCB using adaptive threshold for cutoff values.^17^ All scan readers were blinded to study group, demographics, and time of scan. Inter‐ and intra‐reader variabilities were less than 5%.

### 2.5 Statistical analysis

Data were assessed for normality via skew and kurtosis. Hs‐CRP was log‐transformed to normalize the distribution. Variables were reported as mean ± *SD* for parametric variables, median (interquartile range [IQR]) for nonparametric variables, and percentages (%) for categorical variables. Statistical significance was assessed by Student's *t‐* test for parametric variables, Wilcoxon rank‐sum test for nonparametric variables, and Pearson's *χ* ^2^ test for categorical variables. Uni‐ and multivariate linear regressions were performed and adjusted for hypertension, BMI, low‐density lipoprotein (LDL), triglycerides, and statin intensity. Statin intensity was included to account for differences between participants in each study arm and was calculated as being low/moderate/high based on the 2018 ACC/AHA Classification of Intensity.^18^ Standardized *ß* coefficients were reported, with *p* < .05 considered significant. Regression results were validated via an ANCOVA test. All statistical analyses were performed using StataIC (v. 17.0, StataCorp).

## 3 RESULTS

### 3.1 Effects of inflammation on clinical and lipid parameters

The cohort was middle‐aged (mean ± *SD*; 65 ± 6.3 years), predominantly male (64%), and were overweight according to their BMI (median [IQR]; 27 [25–29]). When stratified by median hs‐CRP at baseline, tobacco use, diabetes prevalence, and total cholesterol levels were not significantly different between groups (Table 1). However, the high hs‐CRP group had increased LDL cholesterol (LDL‐C; 97 [77–113] vs. 87 [75–97] mg/dl; *p* = .01) and triglyceride levels (118 [78–149] vs. 96 [67–121] mg/dl; *p* = .03) than the low hs‐CRP group. There were no significant differences between baseline coronary calcium (CAC) scores or between the change in CAC scores over 2 years across either group (Tables 1 and 4).

### 3.2 Baseline statin intensity of cohort

There were no differences in baseline low/medium/high‐intensity statin use among participants when the cohort was stratified by median value of baseline hs‐CRP (Table 2). The statin intensities were reported according to the 2018 ACC/AHA Classification of Intensity guidelines.^18^

**Table 2:** Statin intensity in cohort stratified by median value of baseline hs‐CRP

Statin intensity^a^	Low hs‐CRP (*n* = 76)	High hs‐CRP (*n* = 66)
Low‐intensity (LDL‐cholesterol reduction <30%)	2 (3)	3 (5)
Moderate‐intensity (LDL‐cholesterol 30% to <50%)	52 (68)	43 (65)
High‐intensity (LDL‐cholesterol reduction >50%)	22 (29)	20 (30)

Abbreviations: hs‐CRP, high‐sensitivity C‐reactive protein; LDL, low‐density lipoprotein.

^a^ Statin intensity reported according to 2018 ACC/AHA guidelines. Values are reported as *N* (%) for categorical data.

### 3.3 Effects of statin treatment on carotid artery wall thickness by MRI

Study participants assigned statin treatment via image‐guided assessment of atherosclerosis via CCTA had a nonsignificant reduction in carotid artery thickness of 3.52 mm^3^ compared to 5.91 mm^3^ in the standard clinical care arm over 2‐year follow‐up (Table S1).

### 3.4 Effects of statins on coronary plaque parameters in those with elevated hs‐CRP

At baseline, the high versus low groups had similar levels of NCB (3.6 [0.1–15.4] mm^2^ × 100 vs. 3.3 [0.4–14.0] mm^2^ × 100; *p* = .99) and DCB (6.1 [0–37.2] mm^2^ × 100 vs. 6.5 [0.5–37] mm^2^ × 100; *p* = .84). Despite these similar levels, participants with high hs‐CRP experienced significant increase in the change in DCB without significant differences in the change in NCB over the 2‐year period of statin treatment (Table 1). Increased hs‐CRP significantly associated with change in DCB and persisted beyond adjustment for hypertension, BMI, LDL‐C, triglycerides, and statin intensity (Table 3). A follow up ANCOVA analysis showed a positive interaction of log of hs‐CRP (log(hs‐CRP)) on the change in DCB beyond adjustment of the same model above [*F*(1,136) = 5.50; *p* = .02]. The change in DCB also displayed an inverse relationship to the change in log(hs‐CRP), which persisted beyond adjustment (Table 3). Finally, the change in CAC scores over 2 years (deltaCAC) was not significantly associated with log(hs‐CRP) or change in log(hs‐CRP) (Table 4).

**Table 3:** Association of change in dense‐calcified coronary burden (DeltaDCB) with log‐transformed high‐sensitivity C‐reactive protein (hs‐CRP) and change in log(hs‐CRP)

DeltaDCB	Standardized *β* a	*p*
Log(hs‐CRP) (Unadjusted)	.191	**.02**
Log(hs‐CRP) (Model 1)	.200	**.03**
Delta Log(hs‐CRP) (Unadjusted)	−.175	**.04**
Delta Log(hs‐CRP) (Model 1)	−.183	**.03**

^a^ Beta is standardized for change in dense‐calcified coronary burden (DCB) Model 1 adjusted for hypertension, body mass index, low‐density lipoprotein, triglycerides, and statin intensity. Bolded *p * values are significant. hs*‐*CRP: high sensitivity C‐reactive protein.

**Table 4:** Association of change in CAC score (DeltaCAC) with log(hs‐CRP) and change in log(hs‐CRP)

DeltaCAC score	Standardized *β* a	*p*
Log(hs‐CRP) (Unadjusted)	.061	.48
Log(hs‐CRP) (Model 1)	.050	.60
Delta Log(hs‐CRP) (Unadjusted)	.010	.91
Delta Log(hs‐CRP) (Model 1)	.0042	.96

^a^ Reported beta is standardized for change in CAC Score. Model 1 adjusted for hypertension, body mass index, low‐density lipoprotein, triglycerides, and statin intensity. Bolded *p * values are significant. CAC score: coronary artery calcification score; hs‐CRP: high‐sensitivity C reactive protein.

## 4 DISCUSSION AND CONCLUSION

**Figure 1:**
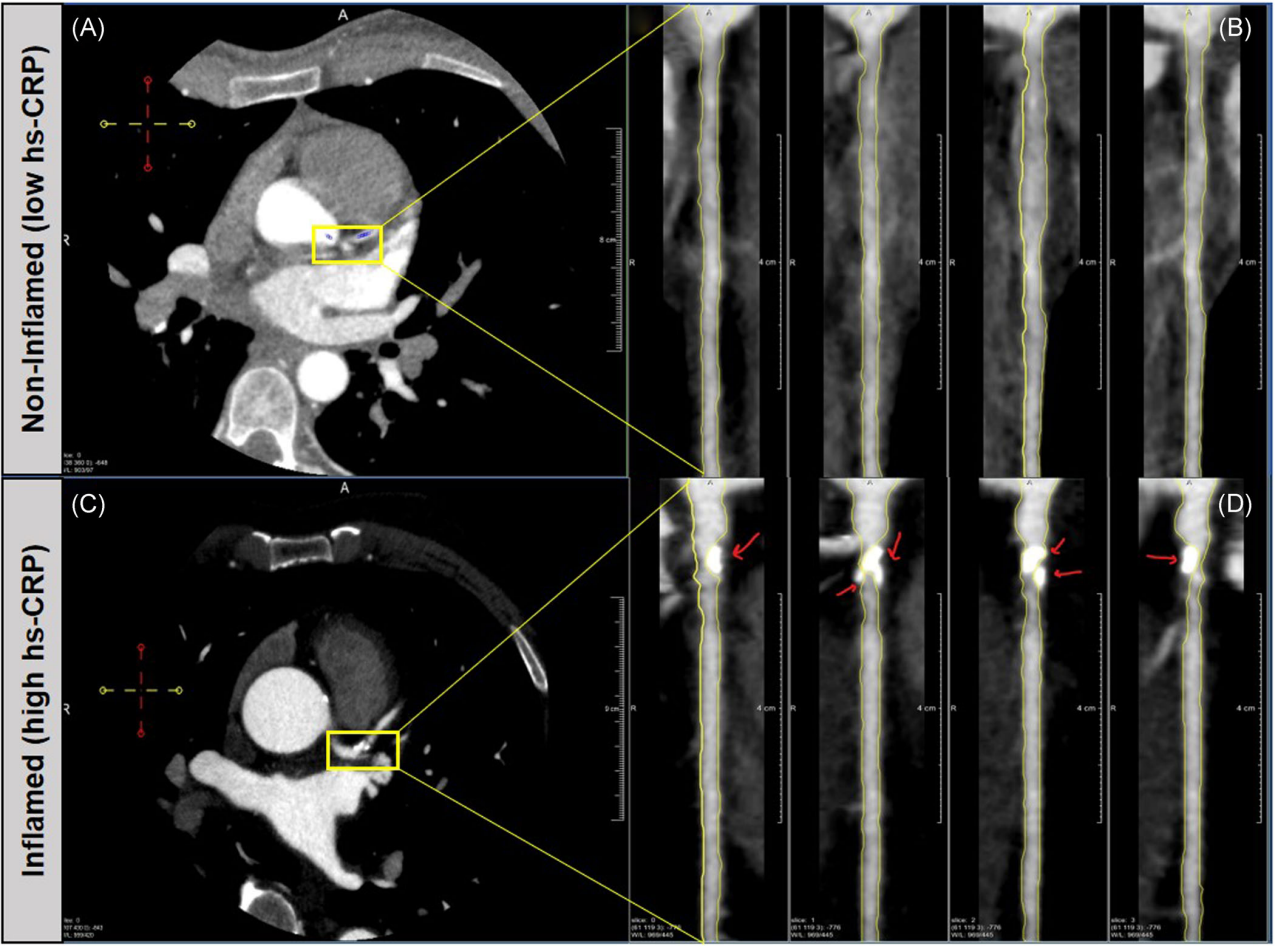
Central illustration of coronary calcification and inflammation. Increased coronary calcification, as measured by dense‐calcified coronary burden, in the left anterior descending artery in patients with higher baseline inflammation, as measured by median high‐sensitivity C‐reactive protein (hs‐CRP). Images were captured through CCTA and analyzed with QAngio CT (Medis). CCTA, cardiac computed tomography angiography

We performed a secondary analysis of the (RIGHT) study (NCT01212900) to assess the potential differential effect of statin treatment on coronary calcification in patients stratified by pretreatment hs‐CRP levels. We found that patients with higher baseline inflammation had significant increase in mean coronary calcification (DCB) progression. Further, baseline hs‐CRP and change in hs‐CRP over 2 years were associated with change in DCB, independent of baseline characteristics and statin intensity. These analyses suggest that the presence of systemic inflammation at baseline before statin treatment is associated with more calcification over 2 years (Figure 1).

In contrast, we did not observe any significant changes in baseline NCB or 2‐year change in NCB progression between these groups. This finding suggests that statin treatment may primarily operate as a method of plaque stabilization rather than as a means of preventing early atherosclerosis in inflamed patients who already have elevated NCB and prevalent atherosclerotic plaque presence. It is possible that patients with greater baseline systemic inflammation may have more local plaque inflammation and elevated plaque lipid content, which leads them to derive more benefit from statin treatment.^19^ Thus, our findings demonstrate the effectiveness of statin treatment in inducing greater coronary calcification in patients with higher baseline inflammation, but larger studies are needed to understand the basis of these findings.

Our results on calcification occurring following statin treatment are in line with the findings from the PARADIGM study (NCT02803411), which characterized the modulation of coronary artery plaque in a primary prevention cohort stratified by statin use^11^ and found that statin treatment associated with slower progression of overall coronary atherosclerosis volume, reduction of high‐risk plaque features, and increased plaque calcification.^11^ The JUPITER study found a reduction in cardiovascular events in those with elevated hs‐CRP after treatment with rosuvastatin^15^ and it is possible that plaque stabilization occurred following statin treatment, but this was not evaluated in that study.

Our findings also suggest the potential contribution of inflammation in promoting microcalcification. Microcalcifications represent an early, active stage of vascular calcification correlated with an inflammatory state and directly contribute to plaque rupture.^20^, ^21^, ^22^ We found that participants with higher baseline inflammation experienced significant increase in coronary calcification over 2 years of statin treatment, as assessed by DCB, illustrating the association between inflammation, microcalcification, and the effect of statin treatment on coronary calcification. However, CAC scores did not differ between high versus low hs‐CRP groups over 2 years. This further confirms that while the CAC score is a good measure of overall plaque burden and stable end‐stage macroscopic calcification, it is not a useful measure in identifying unstable atherosclerotic plaques.^22^, ^23^ Further, our findings suggest that DCB may be a potential sensitive measure of microcalcifications; however, these results must be confirmed with 18F‐sodium fluoride (18F‐NaF) positron emission tomography (PET)/CT imaging, as CCTA has limited spatial resolution to detect such changes.^22^, ^24^ ^18^F‐NaF PET/CT imaging is an imaging modality sensitive to microcalcifications that has been established as a method to better elucidate the relationship between inflammation, microcalcification, and atherosclerotic plaque activity.^22^, ^24^

While our findings provide insight into the potential effects of statins on patients with higher inflammation, we acknowledge that there are limitations to our study. As this study was observational and cross‐sectional in nature, causality and directionality are difficult to establish. It is also a post‐hoc analysis and is therefore subject to inherent residual confounding. Additionally, due to a small sample size, the study groups did not have the power to adjust for additional potential time‐varying covariates, such as changes in hypertension status, BMI, cardiovascular events, or other lifestyle factors. Nonetheless, this study provides serial plaque progression analysis in a single group of study participants over a 2‐year period on the same imaging analyzer and operator, thus minimizing variability.

In conclusion, individuals with a higher baseline inflammation, as assessed by hs‐CRP, had significant increase in coronary calcification following a 2‐year regiment of statin treatment. These results point to a potential increased benefit of statin treatment in patients with high systemic inflammation. Efforts to understand the relationship between inflammation, coronary calcification, and statin treatment are necessary to inform clinicians of the potential benefits of statin treatment, particularly for those affected by inflammatory conditions, and to reduce associated cardiovascular disease risk.

## CONFLICT OF INTERESTS

Dr. Mehta is a full‐time US government employee and has served as a consultant for Amgen, Eli Lilly, and Leo Pharma receiving grants/other payments; as a principal investigator and/or investigator for AbbVie, Celgene, Janssen Pharmaceuticals, Inc, and Novartis receiving grants and/or research funding; and as a principal investigator for the National Institute of Health receiving grants and/or research funding. Dr. Bluemke is the Editor‐in‐Chief of Radiology, a publication of the Radiology Society of North America. All other authors declare no conflicts of interest in relation to the work presented in this manuscript.

## Supporting information

Supporting information.

## Data Availability

The data that support the findings of this study were fully accessed by the lead authors who attest to the responsibility for the integrity of the data analyses. The data are available from the corresponding author upon reasonable request.
