# Supplementary material for: Inflammation, coronary plaque progression, and statin use: A secondary analysis of the Risk Stratification with Image Guidance of HMG CoA Reductase Inhibitor Therapy (RIGHT) study
Source: Clin Cardiol. 2022 Apr 2;45(6):622–8. doi: 10.1002/clc.23808 (PMC9175258; doi:10.1002/clc.23808)
Supplement: Supplementary file 1 — Supporting information. [file CLC-45-622-s001.docx]

**Supplemental Figure 1: Recruitment Scheme**

CONSORT diagram of the recruitment scheme for the cohort denoting patient enrollment and study completion

**Supplemental Figure** [**1**](#MEP_L_fig1)
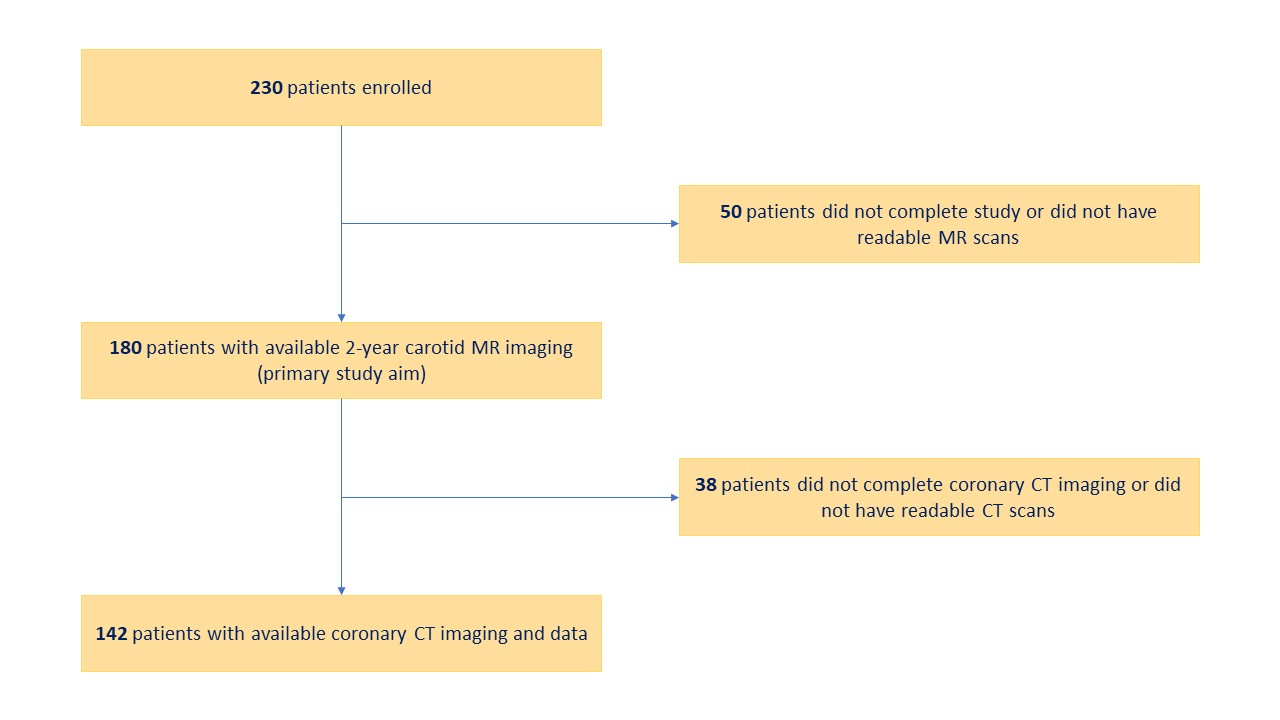


**Supplemental Table 1: Change in carotid artery wall thickness**

|  | **Imaging Arm** | **Standard arm** |
| --- | --- | --- |
|  | **(n**▒=▒**91)** | **(n**▒=▒**89)** |
| **Carotid Artery Thickness (mm^3^)** | -3.52 | -5.91 |
| **Confidence Interval, 95%** | (-7.55 to 0.51) | (-10.97 to -0.85) |
